# Canine Tick-Borne Encephalitis: Clinical Features, Survival Rate and Neurological Sequelae: A Retrospective Study of 54 Cases (1999–2016)

**DOI:** 10.3389/fvets.2021.782044

**Published:** 2021-11-10

**Authors:** Christina Kleeb, Lorenzo Golini, Katrin Beckmann, Paul Torgerson, Frank Steffen

**Affiliations:** ^1^Section of Neurology and Neurosurgery, Small Animal Clinic, Vetsuisse Faculty, University of Zurich, Zurich, Switzerland; ^2^Marigin Tierarztpraxis Farnenbüel, Eschenbach, Switzerland; ^3^Section of Veterinary Epidemiology, Vetsuisse Faculty, University of Zurich, Zurich, Switzerland

**Keywords:** dog, TBE, meningoencephalomyelitis, outcome, cervical weakness, neurological sequelae

## Abstract

Tick-borne encephalitis (TBE) is one of the most important infectious diseases of the central nervous system in dogs from endemic areas. While in humans survival rate and long-term outcomes are well described, these data are lacking in veterinary literature. The aim of the present paper is to characterize the clinical aspects of TBE and to investigate fatality rate, long-term outcome and the long-term neurological sequelae in a population of dogs infected with TBE. We performed a retrospective analysis of 54 dogs diagnosed with TBE at the veterinary hospital of the University of Zurich between 1999 and 2016. Medical data such as signalment, clinical presentation, results of diagnostic procedures, treatment and outcome were collected and analyzed. Statistical analysis including a cox proportional hazard model using a backward stepwise regression approach was performed. In 62% of the TBE cases unspecific signs were described before the onset of neurological signs, resembling a biphasic appearance that is well known in human TBE. Case fatality rate was 33% and all dogs died within the first 4 months after diagnosis. Long-term neurological sequalae were detected in 17% of the TBE cases. For each day of clinical signs before hospital entry the odds of sequalae increased by a factor of 1.88 (CI 1.04–3.15). Older dogs and dogs presented with seizure activity had an increased hazard risk of death (Hazard ration = 1.2, *p* = 0.03; and 9.38, *p* = 0.001, respectively). In conclusion, despite TBE being a life-threatening disease with severe clinical signs, the survival rate in our study was 67%. However, long-term sequalae can be of concern especially in dogs with longer clinical course.

## Introduction

Tick-borne encephalitis (TBE) is a viral disease, endemic in Europe, causing mainly nonspecific febrile illness followed by a remission phase in people. After this initial phase the disease may affect the nervous system in up to 50% of the cases. In approximately 30% to 80% of recovered patients, long-term neurological sequelae are observed (1–4). The disease is caused by the European subtype of Flavivirus (TBEV), a single positive stranded RNA virus, that infects dogs, horses, domestic and wild ruminants, rodents, wild boars, and humans. In Eurasia, TBE has not only been associated with this subtype but also with the Siberian and the Far-Eastern subtypes that tends to show a mono-phasic pattern (1, 2). The TBEV is mainly inoculated by *Ixodes* spp. (4, 5) and *Dermacentor* spp. (6) ticks in Europe. The prevalence of TBEV within the tick population ranges from 0.1 to 5% in the European countries (7, 8). In Switzerland, the prevalence is around 0.46% even in previously considered safe regions such as the southern site of the Alps (8). Epidemiologically, TBE is endemic in 27 countries with a 400% increase of morbidity between 1974 and 2003 in Europe (1). In Switzerland, TBEV is causing over 100 reported human cases annually. In 2020, 455 cases were recorded (9).

The overall TBEV seroprevalence in canines varies but has been estimated in a recent metanalysis to range from 0 to 53.6% in dogs with neurological signs (10). Due to continuous climate changes and international transportation, TBEV should be included in the diagnostic workup of animals from previously unaffected areas such as Ticino (8) or United Kingdom where two autochthonous cases have been recently described (11). Despite canine TBEV infection having a calculated annual risk around 11.6% (12), dogs are rarely clinically affected (13, 14). TBEV was firstly isolated from the brain of a dog with neurological signs in 1972 (15). Since then various authors described TBEV as causes of central (13) and peripheral (16) neurological syndromes in dogs.

To clinically diagnose TBE in canines, we face the same challenges encountered in human medicine. In both species, diagnosis is based upon seasonal occurrence, known recent exposure to ticks, clinical and neurological signs, serological and cerebrospinal fluid (CSF) presence of IgM and/or IgG, and presence of magnetic resonance imaging findings suggestive of a viral encephalopathy (3, 17–20).

Causal treatment is not definitive in both canine and human patients, and acaricidal treatment might not necessary fully prevent infection (12). Therefore, preventive vaccination of the host is of paramount importance although at time of writing this is only available in humans (21).

In human TBE due to the European subtype, fatalities rate up to 2% (3, 19) and neurological sequelae are evident in about 10% of survivors (1, 3, 17–20). Up to date, data about survival and long-term neurological sequelae in dogs with TBE are scanty and not readily available. In previous publications mortality has been reported to be extremely high and TBE was considered to be a fatal neurological disease although based upon only five dogs (22). The aim of the present paper is to characterize the clinical aspects and to investigate case fatality rate, long-term outcome, and to describe long-term neurological sequelae in a clinical population of dogs with TBE presented at our hospital in the years 1999–2016.

## Materials and Methods

### Study Population and Inclusion Criteria

This retrospective observational study was performed searching the database of the Vetsuisse Faculty, University of Zurich, for dogs with a diagnosis of TBE between the years 1999 and 2016. The inclusion criteria were as follows: (1) dogs presented with neurological signs suggestive of central nervous system disease and living in endemic areas for tick-borne encephalitis virus (or coming from an endemic area); (2) cerebrospinal fluid changes compatible with inflammatory disease; and (3) positive antibody titers in cerebrospinal fluid at time of admission (TBEV cerebrospinal fluid titer using ELISA from an external laboratory [Alomed Randolfzell–Böhringen, Deutschland] higher than 2 UI/L). Dogs that did not have positive CSF antibodies at initial presentation were excluded.

Neurological signs were grouped according to previous data on human cases of TBE on meningoencephalitis, encephalomeningomyelitis, meningomyelitis with or without poliomyelitic form, or meningitis (1). This allowed comparison with human TBE. Results of cerebrospinal fluid analysis (including total protein content, total nucleated cell count, and differential cell count) and results of enzyme-linked immunosorbent assay from CSF and/or histopathology in case of euthanasia were reviewed. TBEV detection via PCR of cerebrospinal fluid was performed at our laboratory using real time quantitative PCR (RT-qPCR) technique following the protocol previously published by Wicki and colleagues (23). Hematology and biochemistry results as well as imaging results (radiographs and advanced cross-sectional imaging) were reviewed, when available. Any short-term follow-up information (neurological follow-up examination, telephone follow-up) was included to document sequelae of TBE. A long-term follow-up was performed via structured phone interviews conducted by one of the first authors (CK) with the owners to determine if patients were still alive, to describe the cause of death or request for euthanasia, and if any long-term neurological sequelae were observed by the owners since the diagnosis of TBE. Specifically, gate and/or posture abnormalities, behavioral abnormalities compared to pre TBEV infection, time to fully recover from the neurological syndrome, and persistency of clinical symptoms were investigated.

### Statistical Analysis

All analyses were undertaken in R including descriptive analysis of the demographic data (24). The association between the risk of death and demographic data and the occurrence of different variables (seizures, long-term sequelae, age of dog, TBEV titer, presence of vestibular signs, number of leucocytes in the cerebrospinal fluid, paresis/plegia and type of encephalitis) was assessed with a Cox proportional hazard model using a backward stepwise regression approach. This survival model was used to assess the association of these factors with survival time. The significance level considered was 5% (*P* < 0.05).

The hospitalization times of dogs and time taken until full recovery was analyzed by a gamma generalized linear models (GLM) as the dependent variable was not normally distributed and the gamma model gave the best fit. Analysis of residuals confirmed that the gamma GLM was an appropriate statistical model. For the analysis of time taken to full recovery, dogs that did not achieve recovery were removed from the data set. The risk of developing sequelae was analyzed by a binomial GLM (logistic regression).

## Results

Between 1999 and 2016, 57 dogs were diagnosed with TBE. In three cases, TBEV titer was negative at admission and they were therefore excluded. TBE was later confirmed in these cases, either by repetition of TBEV titers or histopathology.

The remaining 54 cases were presented between March and October and the owners recalled recent tick exposure in 23 of 54 dogs (42.5%). The most commonly affected breeds observed in this population were Newfoundland dogs (8/54), Rottweilers (8/54), and Labrador Retriever (5/54). These breeds accounted for 38.8% of the total population. The mean age at presentation was 5.61 (range 1–13 years). Sex and sexual physiological status were 28/54 males (intact/neutered 20/8) and 26/54 females (intact/neutered 10/16).

### Clinical and Neurological Presentation

Mean duration of clinical signs before admission was 4 days (range: 0–14 days). In 34 of the 54 cases (62%), interviewed owners or first opinion veterinarians reported unspecific signs such as apathy, inappetence, gastrointestinal signs, and hyperthermia 1–10 days before hospital admission. Of these 34 dogs, 23 were reported to have had hyperthermia (23/34, 67.6%) and 15 gastrointestinal signs (15/34, 44.11%).

Physical examination at admission showed hyperthermia (mean 39.9 C; range: 39.1–41.3) in 36 dogs (66.6%). The most common neurological presenting signs at admission included ataxia/vestibular signs (28 dogs, 51.8%), cervical pain (21 dogs, 38.8%), plegia/paresis of one or more limbs (15 dogs, 27.7%), cranial nerve deficits (16 dog, 29.6%), proprioceptive ataxia (16 dogs, 29%), and cervical weakness (11 dogs, 20%). Isolated seizures were recorded in 8 dogs (14.8%) at time of admission whereas two dogs were admitted with a history of status epilepticus.

The clinical neurological syndrome at presentation was consistent with meningitis (3 dogs, 5%), meningoencephalitis (23 dogs, 42%), meningomyelitis (18 dogs, 33%), and meningoencephalo-myelitis (10 dogs/ 19%). Ten subjects showed seizure as part of initial presentation while seven dogs developed them during hospitalization (17 dogs; 31%). Of these 17 dogs, cluster seizures occurred in six (35%), four had focal seizures (23%), four had status epilepticus (23%), and three had isolated epileptic seizures (17%).

### Laboratory Findings

Blood works results were abnormal in 48 cases [non-regenerative anemia (30/48, 62%), neutrophilic leukocytosis (24/48, 53%), and lymphopenia (17/48, 42%)]. The most frequently observed abnormalities in biochemical panel were elevated liver enzymes (24/48, 50%), elevated creatinine kinase (CK) (12/48, 25%), elevated urea (8/48,16%), hyperproteinemia (8/48, 16%), and hypoalbuminemia (6/48, 12%).

CSF analysis showed mean total nucleated cell count of 113.8 cells pro microliter (from 5.5 to 880 WBC per microliter, normal reference range <4 WBC per microliter) and mean protein content of 68.7 mg/dL (ranging from 18 to 180 mg/dL, and a normal reference range <35 mg/dL). TBEV antibody titer in the cerebrospinal fluid was 82.1 UI/L (ranging from 8 to 173 UI/L). A TBEV real time quantitative PCR (RT-qPCR) was performed in 8 cases resulting in 7 negative (7/8, 88%) and 1 positive case (1/8, 12%). Other pathogens (Distemper, Toxoplasma spp., Neospora spp.) were tested by PCR and were negative in all cases. The differential cell count showed lymphocytic pleocytosis (36/54, 66.5%), mononuclear pleocytosis (10/54, 18.5%), or mixed pleocytosis (8/54, 14%).

### Diagnostic Imaging

Radiological studies were obtained in 31/54 (57.4%) dogs. Thoracic radiographs were available in 18 (18/31, 58.06%) and abdominal radiographs in nine (9/31, 29.03%) dogs. A bronchial pattern was seen in three dogs, and atelectasis in two. Otherwise the studies were reported as normal.

In 22/54 (40.7%) cases imaging of the central nervous system was performed with computed tomography (CT) of the brain in 4/22 (18.18%) and MRI of the brain and/or spine in 18/22 (81.81%). MRI findings of 14 dogs have been previously described in Beckmann et al. (25) and Sievert et al. (26). Results of brain CT were within normal limits in all cases. Brain magnetic resonance imaging was carried out on 17 patients and 11 showed abnormal findings (64.7%). In six dogs the findings were confined to the gray matter of the thalamus (6/11, 54.54%) while in others, hippocampus (2/11, 18.18%), pons (1/11, 9.09%), brain stem (3/11, 27.27%), and basal nuclei (1/11, 9.09%) were also affected. The images were consistently similar, symmetric, and hyperintense lesions in T2-weighted sequences compared to white matter, iso- to hypointense in T1-weighted, non-enhancing post contrast administration, and had minimal or no mass effect or perilesional edema. Four of 17 (23.52%) dogs had meningeal enhancement. MRI of the spine performed in 11 cases (11/18, 61.11%) showed abnormal T2WI hyperintensity confined to the ventral horns of the gray matter in two patients (2/11, 18.18%) without post gadolinium administration T1WI contrast enhancement.

### Hospitalization Time and Neurological Status/Outcome at Discharge

The mean hospitalization time was 8 days (range from 1 to 36 days). Tetraplegic cases were hospitalized for an average 10 days (range: 8–12 days); tetraparetic cases 13.7 days (range: 2–35 days); and paraparetic cases 8.7 days (range: 6–12 days).

### Medical Management During Hospitalization

Phenobarbital was the first line medication in all patients with seizures. In 64% (11/17) seizure control was inadequate and Levetiracetam was introduced as a second antiepileptic drug. In those dogs with cluster seizures or status epilepticus constant rate infusions with midazolam was added to control the seizure activity.

In 49/54 cases (90%) anti-inflammatory doses of corticosteroids (dose ranging from 0.5 to 1 mg/Kg/q24h) for 2–5 days were administered, 45 (45/54, 83%) had antibiotics (clindamycin 11 mg/Kg/q12h) pending CSF results.

Fluid therapy, rehabilitation therapy, and nursing care were administered according to patient needs.

### Mortality and Survival Rate

One-third (18/54, 33%) of the dogs in this study died or were euthanized because of complications associated with TBEV infection within 4 months after discharge.

Ten dogs did not survive until discharge or were euthanized within 3 days after discharge (10/54, 18.5%). Six dogs were euthanized on request of the owners because of absent improvement of tetraplegia/tetraparesis (6/10, 60%) during hospitalization (range: 3–18 days), one because of recurring seizures unresponsive to treatment (1/10, 10%), two died spontaneously due to cardiopulmonary arrest (2/10, 20%), and one was euthanized because of concurrent bone tumor (1/10, 10%).

### Long-Term Outcome and Neurological Sequelae

Forty-one of 54 (81%) dogs were discharged from the hospital and survived more than 3 days after discharge. Of those 41 dogs, dogs with non-ambulatory paraparesis (4/41, 9%) were able to ambulate unassisted within 6 days (ranging from 2 to 12 days), whereas tetraplegic/paretic dogs (10/41, 24%) regained ambulation within 12 days (3–26 days). In nine dogs with a cervical weakness (poliomyelitic form), the weakness resolved in all patients within 2–71 days.

In total, three cases were lost for follow-up 30 days after discharge. Of these three dogs, one was neurologically normal (30 days), one had persisting thoracic limb lameness (28 days), and one had generalized proprioceptive deficits and not further well specified behavioral changes (21 days).

Further follow-up interviews were available for 38 cases, while 6 cases were lost for follow-up interviews. The interview was performed 4 months−12 years after hospital discharge.

Six of 38 (15%) dogs were euthanized due to incomplete neurological improvement and owner's perceived poor quality of life within an average of 43.4 days (range: 3–120 days), while 2 cases were euthanized due to unrelated disease within 120 days. Case fatality rate after discharge within the first 4 months was 21% (8/38). No dogs were euthanized afterward because of TBE sequalae. Fifteen dogs of 36 that survived showed complete neurological recovery (15/36, 41%), while 17/36 (59%) dogs had neurologic deficits at the time of writing. In 4 cases (4/38, 11%) no information regarding neurological recovery was available. Reported neurological sequalae included facial palsy (1/17, 6%), weakness in the hind limb (1/17, 6%), and rarely occurring fine head tremors (1/17, 6%). In 4 cases, owners did not report neurological sequalae but asthenia (4/17, 24%). In one case, late occurrence (3 years after discharge) of aggressive behavior was reported during the follow-up phone call but because of lacking neurological reevaluation and behavioral examination a direct correlation with the previous infection cannot be drawn.

Five of 17 dogs with seizures lived longer than 3 months after discharge (5/17, 29.4%). One dog started seizing 1 year after discharge; the owner refused additional workup to assess the possibility of a post infection encephalopathy.

### Necropsy Findings

Necropsy was available in seven dogs. Macroscopic examination of the central nervous system showed meningeal hyperemia in one dog on seven examined. In two dogs a chronic meningitis was evident. Histologically the main finding was consistent with moderate to severe multifocal sterile lymphohystiocitic (4/7) or lymphoplasmacytic (3/7) meningoencephalitis also with spinal cord involvement (poliomyelitis) in 5/7 cases. Furthermore, neuronophagia (7/7), perivascular cuffs (2/7), activated microglia expressed as glial nodules in gray (7/7) and white matter (3/7), diffuse demyelination (2/7) and white matter oedema (1/7). The lesions were affecting the gray matter of the cerebral cortex (7/7), the hippocampus (7/7), the basal ganglia (6/7), the thalamus (7/7), the brainstem (7/7), the cerebellum (7/7). The spinal cord was affected by a poliomalacia in 5/7 cases, and in 4 of them the lesions were widespread along the entire neuroaxis, while in one the cervicothoracic region was mainly affected. In the latter, the ventral rootlets of the cervicothoracic intumescence were also investigated revealing presence of multifocal axonal degeneration and Schwann cells proliferation. In three cases immunohistochemistry for TBEV was performed confirming clinical diagnosis with positivity in glial nodules (3/3) and ventral horn axons (1/3).

### Statistical Analysis

Six cases lost for follow-up after discharge were excluded from further survival analysis. Our statistical analysis of the reported data showed that dogs with increased temperature at admission (>39.0) dogs with tetraplegia or tetraparesis, and dogs showing signs of recovers survived longer (Table 1). In contrast dogs with seizures had a shorter survival time. Survival time also decreased with age.

**Table 1:** Factors influencing survival of dogs diagnosed with tick born encephalitis.

**Factor**	Hazard ratio^*^	***P*-value**
Age^∧^	1.2 (1.04–1.44)	0.013
Presence of seizures	9.38 (2.43–36.14)	0.001
Presence of fever	0.22 (0.06–0.78)	0.02
Presence of tetraplegia/tetraparesis	0.16 (0.03–0.71)	0.02
Recovery	0.005 (0.0004–0.05)	<0.0001

* *Hazard ratio >1 indicates an increased risk of death in a time period and hence shorter survival*.

∧ *Age is a continuous variable, so hazard ratio is for each 1 year increase in age. Other variables are binomial and the hazard ratio is the ratio between the presence and absence of the factor*.

Dogs presenting with fever or tetraplegia/tetraparesis due to meningoencephalomyelitis and meningomyelitis/poliomyelitic tended to have a longer hospitalization time. Dogs presenting with signs of tetraplegia/tetraparesis took 1.32 (CI 1.05–1.80) times longer compared to others, to regain normal ambulation. The odds of developing sequelae increased with the duration of clinical signs before admission. For each day of clinical signs before hospital entry the odds of sequalae increased by a factor of 1.88 (CI 1.04–3.15). Although neurological sequalae are various, the hazard model did not reveal any negative outcome associated with these sequalae (Table 1).

## Discussion

This paper presents a population-based study about overall clinical presentation, clinical course, fatality rate, and neurological sequelae of TBEV infection in large population of affected dogs. Previously, this information could be only inferred from different publications, encompassing case reports or small case series published from 1972 to 2014, ranging from one to a maximum 12 dogs affected by TBEV (22, 27, 28).

In humans, European TEBV infection shows typically a biphasic appearance (1–3), and during the initial consultation unspecific symptoms such as fever, malaise, headache, sickness, vomiting, and muscular pain are reported up to 10 days before neurological signs occur (1–3, 17). In dogs a biphasic disease-course has been debated controversially (27). In this study population 34 owners or referring veterinarians recalled fever, and unspecific GI signs occurring from 1 to 10 days before the admission at our referral center. This supports that “malaise” as first clinical signs of TBEV viremia does occur in dogs as well.

Hyperthermia was evident in two-thirds (36/54) of our population and interestingly dogs with hyperthermia had a significantly lower risk to die compared to those that did not show hyperthermia. Hyperthermia is a hallmark of active infection and quite common in acute viral encephalitis. Non-structural proteins of TBEV antagonize type 1 interferon signaling (29) suppressing the Janus kinase-signal transducer and activator of transcription (JAK-STAT) pathway (2). This inhibition of type 1 interferon is causing a reduced anti-viral immunity in the host. Possibly patients with higher flavivirus load have a stronger suppression of type 1 interferon signaling, and therefore are not showing fever which may possibly influence outcome negatively (29).

Clinical signs of TBE are variable in humans and dogs (1, 19). In humans, the predominant form of TBE is meningitis (1, 19) while in the dogs in our study meningitis only was a rare presentation. Possibly dogs with meningitis only, and therefore milder clinical signs, are less likely to be referred. In contrast to humans, meningomyelitis and meningoencephalo-myelitis (1, 19) are common in dogs. TBE, because of its predilection to affect large neurons, frequently causes poliomyelitis (3, 18). Poliomyelitis primarily presenting with neck weakness is uncommon in other etiologies of myelitis in dogs (28, 30), and mainly reported in viral myelitis (16, 31). Similar to human meningoencephalitis it is also common in dogs. While seizures are a sporadic event in human TBE, being reported in up to 3% of the cases only, in dogs seizures occurred in 31% of the cases.

The hematobiochemical changes are compatible with an acute systemic inflammation, and similar to those described in human medicine (17, 32), however it would be interesting in the future to investigate positive acute phase proteins in active acute TBE, especially since hypoalbuminemia and hyperglobulinemia were a common feature in our population.

Cross sectional imaging is of utmost importance for neurological diagnosis. While CT was not useful in detecting the cause of neurological signs in TBE (17), MRI showed abnormal findings supportive of TBEV infection in two-thirds of our population. As previously published in human and veterinary literature, these findings are also not pathognomonic for TBE (1, 16, 17, 25, 26).

Diagnosis of TBE, especially in cases with normal MRI, can be challenging (1, 3, 8, 17). We had to exclude 3 cases from our study because of negative antibody titers in CSF at presentation. In these cases, most likely the clinical signs developed so rapidly that CSF antibodies against TBEV were initially negative, despite showing clinical signs compatible with TBE. However, in all of these three dogs TBE was later confirmed, two being tested positive for TBEV in CSF 1 and 2 weeks after initial presentation, respectively, and in the third case by positive immunohistochemical staining of brain specimen, acquired during necroscopy. Also, in human medicine it is known that TBEV could sporadically determine hyperacute neurological syndromes without CSF alterations (33) or initial negative antibodies testing (1–3, 17). In these cases, the gold standard is seroneutralization, but this is an expensive test requiring live virus, and flavivirus can be handled only by specific laboratories. For diagnostic purpose, positivity in commercial IFA or ELISA kits in the CSF has been used in the present paper in accordance to human standards as diagnostic hallmark of TBEV infection in presence of consistent clinical signs (1, 3, 17, 19), even with normal CSF examination (33). In dogs it has been shown that commercial ELISA kits have a specificity of 98.2% thus performing well as a diagnostic kit for dogs from endemic areas, such as ours (34). In our population, the magnitude of positivity was not a predictor of a negative outcome.

A RT-qPCR evaluation of CSF for viral RNA has been tempted unsuccessfully in 88% of cases in this study, and it could be explained because viral clearance is a fast process in TBE; TBEV RNA could be detected only up to 3 days from neuroinvasion (19, 27). Moreover, our assay, in comparison to a nested RT-qPCR previously described by Hekrlová et al. (35), does not perform the second nested PCR amplification, thus possibly reducing sensitivity in detecting viral RNA but, on the other hand, it is less prone to false positive results due to possible contaminations during the two-step procedure.

Pathological findings were available for review in only seven patients. In our population, the main findings were a widespread meningoencephalopoliomyelitis in five out of seven patients and three dogs were also positive for TBEV at immunohistochemistry within glial nodules (3/3) and also within axons (1/3). In one case, also, severe changes within ventral rootlets characterized by axonal degeneration and Schwann cells were observed, supporting the neurotropism of this virus mainly for large motoneurons (2, 13, 15, 27).

Treatmentwise, TBE in humans and in dogs require intense nursing care and only recently some antiviral agents have been used in humans without reaching a general consensus about efficacy (1, 3, 17). Corticosteroids have been used in our patients to decrease the brain inflammation, pending results about infectious disease testing. In our practice a short course of steroids has been routinely used in dogs diagnosed with inflammatory central nervous disease with the intention to limit severe neuroinflammation. However, because the lack of untreated controls we could not assess the benefit of a short course of corticosteroids on outcome. In human medicine, neither steroids nor osmoactive drugs, such as mannitol, have been advocated for use on TBE patients, whereas pharmacologic induced coma (deepening of analgosedation) followed as second line by therapeutic hypothermia, or decompressive craniotomies have been recommended (3, 17). Therefore, therapeutic strategies in dogs need to be further studied before robust treatment recommendations can be formulated.

In humans, survival of the neurological infection is not uncommon, however long-term sequelae should be expected in up to 10% of the patients ranging from cognitive domain (memory impairment) or psychiatric (depression) to motor and sensory deficits because of poliomyelopathy or polyradiculoneuropathy (3, 17–19, 36). In a recent paper, sequalae were observed more frequently with clinical signs compatible with meningoencephalomyelitis or meningoencephalitis, respectively (in 43 and 25% of patients); meningitis resulted in fewer sequelae (12.6%) (36).

Little is known about long-term sequelae in dogs affected by TBE. In the present study, long-term sequelae were identified in 17% of the cases. These should raise the awareness of long-term neurological sequelae in dogs. In human patients more severe clinical syndromes (meningoecephalitis, meningoencephalomyelitis/poliomyelitic) are more likely to results in long-term signs (1, 18, 19, 36). Dogs in our study had a significantly increased risk for sequelae when they had shown longer clinical signs before admission.

While a possible behavioral sequela was only reported in one of the dogs in our study, motor neurological sequelae were commonly reported. A refinement of neurobehavioural assessment in dogs could reveal an increased frequency of abnormalities.

Previously, a high mortality of TBEV affected dogs compared to humans was reported. In the previously reported cases, fatalities within first weeks of disease ranging from 16 to 50% (27) to 100% (22), in our population 33% of dogs died within 4 months from the diagnosis, without TBE related deaths were recorded after this time.

An increased risk of death was found in dogs with seizures. This is in contrast to dogs with meningoencephalitis of unknown origin in which survival was not affected by the occurrence of seizures (28, 37). Severe seizure types (cluster seizures, status epilepticus) were observed in a majority of dogs and seizures were poorly controlled following administration of first line medication, phenobarbital, with 61% of non-responders explaining the morbidity associated with their occurrence. Nevertheless, seizure as expression of post infection long term sequelae has affected only one of our cases rendering an association with TBE uncertain. Seizures have been recently also described as long-term sequelae in dogs suffering from MEUO (38).

Another aspect of interest highlighted in our population, and not previously reported, was that the hazard risk of death increased with the age of the patient. In other words, older animals were more at risk of death, similar to what has been observed in human patients with TBE (3). An explanatory mechanism is unknown in humans as well as in our patients. However, before corroborating this parallelism, further research about reasons for euthanasia in TBEV infected dogs should be undertaken. In fact, another aspect not yet investigated is also the economic burden that some owners might face. Leister et al. (39) in a study about tick paralysis in cats in Australia, reported that 250/2,077 cats were euthanized due to financial restriction to perform medical treatment. This financial factor has not been evaluated in this or any previous research about short- or long-term outcome in dogs with neurological disease that require long-term hospitalization, also involving intensive care units. Future research will also take this important factor in account. Furthermore, our study highlighted the role of hyperthermia, however due to its retrospective nature, true fever could not be differentiated readily from exercise-induced hyperthermia due to seizure activity. Therefore, we aim to further evaluate those prognostic factors in a prospective study.

Concluding, our study shows many similarities and few differences of TBE between dogs and humans. Despite case fatality rate could reach 33% of the affected population within initial 4 months, survival is commonly observed, but long-term sequalae might be encountered.

## Data Availability Statement

The original contributions presented in the study are included in the article/supplementary materials, further inquiries can be directed to the corresponding author.

## Ethics Statement

Ethical review and approval was not required for the animal study because of the retrospective nature of the study that is based on preexisting medical records filed to achieve full diagnosis. Written informed consent was obtained from the owners for the participation of their animals in this study.

## Author Contributions

LG and FS designed the study. CK collected the data. LG and CK wrote the first draft of the manuscript. PT performed statistical analysis and performed a proof screening for British English language. KB, LG, and FS reviewed and finalized the manuscript. All authors contributed to the article and approved the submitted version.

## Conflict of Interest

The authors declare that the research was conducted in the absence of any commercial or financial relationships that could be construed as a potential conflict of interest.

## Publisher's Note

All claims expressed in this article are solely those of the authors and do not necessarily represent those of their affiliated organizations, or those of the publisher, the editors and the reviewers. Any product that may be evaluated in this article, or claim that may be made by its manufacturer, is not guaranteed or endorsed by the publisher.

## Abbreviations

CI: Confidence Interval
CSF: Cerebrospinal fluid
CT: Computer tomography
ELISA: Enzyme-Linked ImmunoSorbent Assay
GI: GastroIntestinal
GLM: Generalized linear models
JAK-STAT: Janus kinase-signal transducer and activator of transcription
IFA: Indirect fluorescent antibody
IgM: Immunoglobulin M
IgG: Immunoglobulin G
MEUO: MeningoEncephalitis of Unknown Origin
MRI: Magnetic resonance imaging
RNA: Ribonucleic Acid
TBE: Tick-borne Encephalitis
TBEV: Tick-borne Encephalitis Virus
WBC: White Blood Cells.

## References

[1] Riccardi N Antonello RM Luzzati R Zajkowska J Di Bella S Giacobbe DR. Tick-borne encephalitis in Europe: a brief update on epidemiology, diagnosis, prevention, and treatment. Eur J Intern Med. (2019) 62:1–6. 10.1016/j.ejim.2019.01.004 30678880

[2] Yoshii K. Epidemiology and pathological mechanisms of tick-borne encephalitis. J Vet Med Sci. (2019) 81:343–7. 10.1292/jvms.18-0373 30674746 PMC6451894

[3] Lindquist L Vapalahti O. Tick-borne encephalitis. Lancet. (2008) 371:1861–1. 10.1016/S0140-6736(08)60800-4 18514730

[4] Pavlov P. Studies on tickborne encephalites of sheep and their natural foci in Bulgaria. Zentralbl Bakteriol Orig. (1968) 206:360–7. 5751106

[5] Zindel W Wyler R. Zeckenenzephalitis bei einer Ziege im untern Prättigau. Schweiz Arch Tierheilkd. (1983) 125:383–6. 6612291

[6] Karbowiak G Biernat B Werszko J Rychlik L. The transstadial persistence of tick-borne encephalitis virus in Dermacentor reticulatus ticks in natural conditions. Acta Parasitol. (2016) 61:201–3. 10.1515/ap-2016-0028 26751892

[7] Gäumann R Mühlemann K Strasser M Beuret C.M. High-throughput procedure for tick surveys of tick-borne encephalitis virus and its application in a national surveillance study in Switzerland. Appl Environ Microbiol. (2010) 76:4241–9. 10.1128/AEM.00391-10 20453126 PMC2897458

[8] Casati Pagani S Frigerio Malossa S Klaus C Hoffmann D Beretta O Bomio-Pacciorini N. First detection of TBE virus in ticks and sero-reactivity in goats in a non-endemic region in the southern part of Switzerland (Canton of Ticino). Ticks Tick Borne Dis. (2019) 10:868–74. 10.1016/j.ttbdis.2019.04.006 31047827

[9] Bundesamt für Gesundheit. Zeckenenzephalitis FSME. (2021). Available online at: www.bag.admin.ch (accessed September 9, 2021).

[10] Springer A Glass A Topp AK Strube C. Zoonotic Tick-borne pathogens in temperate and cold regions of Europe-a review on the prevalence in domestic animals. Front Vet Sci. (2020) 7:604910. 10.3389/fvets.2020.604910 33363242 PMC7758354

[11] Holding M Dowall S Hewson R. Detection of tick-borne encephalitis virus in the UK. Lancet. (2020) 395:411. 10.1016/S0140-6736(20)30040-4 32035545

[12] Leschnik M Feiler A Duscher GG Joachim A. Effect of owner-controlled acaricidal treatment on tick infestation and immune response to tick-borne pathogens in naturally infested dogs from Eastern Austria. Parasit Vectors. (2013) 6:62. 10.1186/1756-3305-6-62 23497548 PMC3623908

[13] Leschnik MW Kirtz GC Thalhammer JG. Tick-borne encephalitis (TBE) in dogs. Int J Med Microbiol. (2002) 291(Suppl. 33):66–9. 10.1016/S1438-4221(02)80014-5 12141763

[14] Pfeffer M Dobler G. Tick-borne encephalitis virus in dogs–is this an issue? Parasit Vectors. (2011) 13:59. 10.1186/1756-3305-4-59 21489255 PMC3094398

[15] Gresíková M Sekeyová M Weidnerová K Blaskovic D Steck F Wandeler A. Isolation of tick-borne encephalitis virus from the brain of a sick dog in Switzerland. Acta Virol. (1972) 16:88. 4400683

[16] Beckmann K Oevermann A Golini L Steffen F Kircher P Carrera I. MRI findings in a case of canine tick born meningoencephalomyelitis. Schweiz Arch Tierheilkd. (2014) 156:395–9. 10.1024/0036-7281/a000612 25082637

[17] Taba P Schmutzhard E Forsberg P Lutsar I Ljøstad U Mygland Å. EAN consensus review on prevention, diagnosis and management of tick-borne encephalitis. Eur J Neurol. (2017) 24:1214. 10.1111/ene.13356 28762591

[18] Hrnjaković Cvjetković I Cvjetković D Patić A Radovanov J Kovacević G Milosević V. Tick-borne encephalitis virus infection in humans. Med Pregl. (2016) 69:93–8. 10.2298/MPNS1604093H 27506096

[19] Bogovic P Strle F. Tick-borne encephalitis: a review of epidemiology, clinical characteristics, and management. World J Clin Cases. (2015) 3:430–41. 10.12998/wjcc.v3.i5.430 25984517 PMC4419106

[20] Valarcher JF Hägglund S Juremalm M Blomqvist G Renström L Zohari S. Tick-borne encephalitis. Rev Sci Tech. (2015) 34:453–66. 10.20506/rst.34.2.2371 26601448

[21] Salát J Formanová P Hunady M Eyer L Palus M Ruzek D. Development and testing of a new tick-borne encephalitis virus vaccine candidate for veterinary use. Vaccine. (2018) 36:7257–61. 10.1016/j.vaccine.2018.10.034 30337175

[22] Tipold A Fatzer R Holzmann H. Zentraleuropäische Zeckenenzephalitis beim Hund. Kleintierpraxis. (1993) 38:619–28.

[23] Wicki R Sauter P Mettler C Natsch A Enzler T Pusterla N. A swiss army survey to determine the prevalence of Francisella tularensis, members of the Ehrlichia phagocytophila genogroup, Borrelia burgdorferi sensu lato, and tick-borne encephalitis virus in ticks. Eur J Clin Microbiol Inf Dis. (2000) 19:427–32. 10.1007/s100960000283 10947217

[24] R Core Team. R: A Language and Environment for Statistical Computing. R Foundation for Statistical Computing. Vienna (2020). Available online at: http://www.R-project.org

[25] Beckmann K Steffen F Ohlerth S Kircher PR Carrera I. Three tesla magnetic resonance imaging findings in 12 cases of canine central european tick-borne meningoencephalomyelitis. Vet Radiol Ultrasound. (2016) 57:41–8. 10.1111/vru.12303 26466523

[26] Sievert C Richter H Beckmann K Kircher PR Carrera I. Comparison between proton magnetic resonance spectroscopy findings in dogs with tick-borne encephalitis and clinically normal dogs. Vet Radiol Ultrasound. (2017) 58:53–61. 10.1111/vru.12427 27714889

[27] Pfeffer M Schmuck HM Leschnik M. TBE in animals. In: Dobler G, Erber W, Bröker M, Schmitt HJ, editors. The TBE Book, 3rd ed. Singapore: Global Health Press Pte Ltd. (2020). p 105–19.

[28] Mariscoli M Jaggy A. Clinical and electroencephalographic findings of inflammatory and infectious diseases of the central nervous system in dogs: a retrospective study. Zentralbl Veterinarmed B. (1997) 44:1–18. 10.1111/j.1439-0450.1997.tb00945.x 9084229

[29] Thurmond S Wang B Song J Hai R. Suppression of type I interferon signaling by flavivirus NS5. Viruses. (2018) 10:712. 10.3390/v10120712 30558110 PMC6316265

[30] Cornelis I Volk HA Van Ham L De Decker S. Clinical presentation, diagnostic findings and outcome in dogs diagnosed withpresumptive spinal-only meningoen-cephalomyelitis of unknown origin. J Small Anim Pract. (2017) 58:174–82. 10.1111/jsap.12622 28267222 PMC7166691

[31] Green L Cook L Martinez M Green E. Distemper encephalomyelitis presenting with lower motor neuron signs in a young dog. J Am Anim Hosp Assoc. (2020) 56:127–32. 10.5326/JAAHA-MS-6736 31961216

[32] Barp N Trentini A Di Nuzzo M Mondardini V Francavilla E Contini C. Clinical and laboratory findings in tick-borne encephalitis virus infection. Parasite Epidemiol Control. (2020) 10:e00160. 10.1016/j.parepi.2020.e00160 32637663 PMC7327414

[33] Pöschl P Kleiter I Grubwinkler S Bumes E Bogdahn U Dobler G. Schwere Frühsommer-Meningo-Enzephalomyelitis ohne Liquor-Pleozytose. Fortschr Neurol Psychiatr. (2009) 77:591–3. 10.1055/s-0028-1109768 19821222

[34] Klaus C Beer M Saier R Schubert H Bischoff S Süss J. Evaluation of serological tests for detecting tick-borne encephalitis virus (TBEV) antibodies in animals. Berl Munch Tierarztl Wochenschr. (2011) 124:443–9. 10.2476/0005-9466-124-444 22191165

[35] Hekrlová A Kubíček O Lány P Rosenbergová K Schánilec P. Tick-borne encephalitis in dogs: application of “nested real-time RT-PCR” for intravital virus detection. Berl Munch Tierarztl Wochenschr. (2015) 128:397–401. 10.2376/0005-9366-128-397 26591386

[36] Czupryna P Grygorczuk S Krawczuk K Pancewicz S Zajkowska J Dunaj J. Sequelae of tick-borne encephalitis in retrospective analysis of 1072 patients. Epidemiol Infect. (2018) 146:1663–70. 10.1017/S0950268818002005 30047354 PMC9507954

[37] Lowrie M Smith PM Garosi L. Meningoencephalitis of unknown origin: investigation of prognostic factors and outcome using a standard treatment protocol. Vet Rec. (2013) 172:527. 10.1136/vr.101431 23462382

[38] Kaczmarska A José-López R Czopowicz M Lazzerini K Leblond G Stalin C. Postencephalitic epilepsy in dogs with meningoencephalitis of unknown origin: clinical features, risk factors, and long-term outcome. J Vet Intern Med. (2020) 34:808–20. 10.1111/jvim.15687 31990104 PMC7096646

[39] Leister E Morton J Atwell R Webster R. Clinical presentations, treatments and risk factors for mortality in cats with tick paralysis caused by Ixodes holocyclus: 2077 cases (2008–2016). J Feline Med Surg. (2018) 20:465–78. 10.1177/1098612X17733628 28994630 PMC11104066

